# Barriers of conducting and completing research in Pakistan among doctors: A cross-sectional survey

**DOI:** 10.12669/pjms.41.3.10424

**Published:** 2025-03

**Authors:** Zainab Aqeel Khan, Umaimah Muzzamil, Khubab Ahsan, Aiman Khan, Masood Jawaid

**Affiliations:** 1 Zainab Aqeel Khan, DPT, MSBE Assistant Manager, Medical Affairs Department, PharmEvo, 402 Suite, Business Avenue, Shahrah-e-Faisal Block-6 PECHS, Karachi, Pakistan; 2 Umaimah Muzzamil, B. Pharm, MBA General Manager, Medical Affairs Department, PharmEvo, 402 Suite, Business Avenue, Shahrah-e-Faisal Block-6 PECHS, Karachi, Pakistan; 3 Khubab Ahsan, MBBS, Medical officer, Department of Orthopedics, Jinnah Postgraduate Medical Center, Karachi, Pakistan; 4 Aiman Khan, D. Pharm, Senior officer, Medical Affairs Department, PharmEvo, 402 Suite, Business Avenue, Shahrah-e-Faisal Block-6 PECHS, Karachi, Pakistan; 5 Masood Jawaid, MBBS, FCPS, MHPE, Director, Medical Affairs Department, PharmEvo, 402 Suite, Business Avenue, Shahrah-e-Faisal Block-6 PECHS, Karachi, Pakistan

**Keywords:** Research, Physicians, Publications, Research and development, Pakistan

## Abstract

**Objectives:**

To identify the barriers encountered while conducting and completing research among doctors in Pakistan.

**Methods:**

This cross-sectional study was conducted from November 2023 to March 2024 to identify research barriers among medical doctors working in different hospitals of Pakistan. All graduated doctors who at least had six months of postgraduate clinical experience were included. Participants who provided incomplete response or refused to participate were excluded. The data was collected on pre-designed questionnaire. The survey consisted of demographics, information about research experience and publications and perception about research barriers. P-value <0.05 was considered significant.

**Results:**

Out of 1,000 doctors included for study, only 380 of them responded to the survey with a response rate of 38%. The mean age of all participants was 29.3 ± 7.3. Almost 58% of the participants were female. Almost 62.6% of the participants considered lack of training as the main barrier in conducting research, followed by inadequate financial support (53.1%), inadequate support from institute (53.1%), inadequate support from mentors (48.1%), lack of statistical work support (47.3%), insufficient time (45.5%) and difficulty in obtaining ethical approval (30.7%). There was statistically significant association found for inadequate financial support barrier between participants who had research publication and who did not (P < 0.001).

**Conclusion:**

The lack of training and inadequate financial and institutional support is identified as main barriers for conduction and completion of research in Pakistan among doctors.

## INTRODUCTION

Research is crucial part of medical sciences that promote evidence-based practice. It is important for all healthcare professional to conduct good quality research in order to advance medical knowledge. Medical doctors serve as a front-line leader in medical field and can play an essential role to promote research. However, conduction and completion of research is not an easy task and require a lot of support and dedication. Therefore, in order to promote research culture in medicine, the identification of the subsequent barriers is the first step towards promotion of healthy research culture.^1^,^2^

Several studies reported the barriers to conduct research in Western countries. They identified insufficient time, funding and opportunities as leading barriers, which may be even more in developing world.^2^ However, it was a norm in developing countries to follow and relied on research findings from Western world. This was a huge challenge for evidence-based practice in developing part of the world. Therefore, some advancements have been made with time to conduct research in developing countries as well. Nevertheless, the quality of the research projects is still questionable due to barriers and challenges towards research. Most the studies reported insufficient research allotted time, lack of financial, incentives and inadequate statistical support as barrier towards research. Some other studies also revealed lack of skills and cultural factors as research barriers in developing countries.^3^,^4^

Ahmad S et al. reported barriers faced by medical students to conduct and complete research in Pakistan. They revealed that only 6.6% of students had published an article and they reported lack of mentorship, and lack of research teaching as main barriers.^4^ Another study conducted in Peshawar reported lack of interest (16.7%), lack of time (58%), exam phobia (49.3%) and lack of training (31.4%) and research methodology (25.3%) as barriers to conduct research.^5^

In Pakistan, the situation is compounded by additional factors. Despite the major public health concerns like polio, tuberculosis, obesity and others, there is still a significant gap in comprehensive data concerning the involvement of Pakistani doctors in research activities and the specific barriers they face.^4^ Research involving Pakistani undergraduate medical students and junior faculty highlighted specific obstacles such as lack of mentorship, inadequate research training, and limited financial and statistical support.^4^,^5^ Several organizations such as Higher Education Commission (HEC), Pakistan Health Research Council, PharmEvo Research Forum and others are working religiously to promote and advocate research culture in Pakistan. All these efforts will be more high-yield and meaningful with recent evidence about practical challenges and barriers towards research for doctors.^6^-^9^

To address these problems, our study aimed to identify the barriers encountered while conducting research among doctors in Pakistan. To our knowledge, there is limited literature available that address research barrier among doctors in Pakistan. This study illuminates the challenges faced by a wide spectrum of medical doctors, including physicians, surgeons, junior doctors, postgraduate trainees, and consultants who serve as front line in medical profession.

## METHODS

A cross-sectional survey was conducted to assess the barriers encountered by medical doctors in Pakistan regarding research conduction and completion. Participants were recruited from November 2023 to March 2024. Eligible participants were medical doctors with a minimum of six months of work experience were included after taking informed consent.

### Ethical approval:

The Ethical approval was obtained from Ameen Medical and Dental Center Ethical Committee of the reference number Ref#: ERC-CIRS-2023-602, Dated: September 23, 2023.

### Inclusion & Exclusion Criteria:

All specialty and age groups were included in this study. Participants who filled incomplete form or provide any kind of inaccurate information were excluded. The non-probability convenient sampling was used to enroll participants. We disseminated the survey questionnaire to all major hospitals in all metropolitan cities. All of the participants were contacted via email. However, some of them were approached during academic workshops and conferences. The data was collected using a self-designed questionnaire adopted from previous study with some modifications according to study objective.^7^

The questionnaire was distributed among doctors working both in private and public section hospital and clinics, either online via Google Forms or in hard copy where digital methods were impractical. The questionnaire comprised three sections: demographic information and qualifications (Section-A), research experiences (Section-B) like information about research training, previous research involvement, research publications, conference presentations. The last section (Section-C) comprised of questions related to barriers of conducting and completing research using a Likert scale for barrier assessment and an open-ended question for additional barriers.

Prior to the main survey, a pilot study was conducted to identify and rectify any issues with questionnaire comprehension. The identified issues were resolved before starting data collection for the survey. The Cronbach Alpha value was 0.76, ensuring its internal consistency. None of the participants had difficulty understanding the questionnaire in English. Therefore, we used the English version of the questionnaire to ensure accuracy.

The sample size was calculated using the Open Epi sample size calculator, based on previous findings that 41.5% of junior faculty in Pakistani medical universities were involved in research.^8^ With a 95% confidence interval, 5% margin of error, and 80% power, the minimum required sample size was determined to be 373 participants.

### Statistical analysis:

The data was analyzed by using Statistical Package for the Social Sciences (SPSS) version 26. Descriptive statistics were used to summarize the results. The continuous variables like age, years of experience, number of research training, research published, research presentation and research abandoned were reported in mean and standard deviation (SD) and ranges. Frequencies and percentages were used for other categorical variables. The chi-square test was analyzed to find out the associations between research publications and barriers. P-value < 0.05 were considered significant.

## RESULTS

Out of one thousand doctors included in the study, only 380 of them responded to the survey with a response rate of 38%. The mean age of all participants was 29.3 ± 7.3. Almost 58% of the participants were female. Most of the doctors (39.7%) were house officers followed by postgraduate trainees (34.2%) and registrars (11.5%). A total of 278 (73%) doctors were working in public hospitals. The most common areas of specialization among participants were general medicine (28.7%), general surgery (20%) and orthopedics (13.7%). The mean years of experience was 4.3 ± 5.8. All demographic details of study participants are presented in Table-I.

**Table-I:** General Demographics of participants

Characteristics	Participants (N= 380)
Age (in years)	29.3 ± 7.3
***Gender***	
Male	159 (41.8%)
Female	221 (58.2%)
***Designation***	
Professor	4 (1)
Associate Professor	5 (1.3)
Assistant professor	21 (5.5)
Consultant	20 (5.2)
Registrar	44 (11.5)
Postgraduate trainee	130 (34.2)
House officer	151 (39.7)
Lecturer	5 (1.3)
***Type of hospital***	
Public	278 (73.2)
Private	68 (17.9)
Both	34 (8.9)
***Specialty***	
General medicine	109 (28.7)
General surgery	76 (20)
Orthopaedics	52 (13.7)
Gynaecology	26 (6.8)
Oncology	15 (3.9)
Neuro-medicine	13 (3.4)
Plastic surgery	13 (3.4)
Other	76 (20)
***Years of experience (in years)***	4.3 ± 5.8

Only 98 (25.8%) of the participants had research publications with median (IQR) of 2.50 (2) number of publications. Regarding research experiences, approximately 178 (46.8%) of the participants had a formal research training with 3 (2) number of trainings. Participants who had a formal research training 43.8% of the participants published research papers (P < 0.01). Similarly, 175 (46.1%) of all participants were previously involved in research projects. Involved projects of 71% were observational studies. Among them, 25.3% of the participants published research papers with statistically significant difference (P < 0.01). Almost 71 (18.7%) of the total participants presented their researches in a conference with median of 1.5± 2 research presentations. Among them, 54 (14.2%) published their research papers. There was a statistically significant difference found in research publication between participants who presented in conference with those who did not (P= <0.01). Approximately, 15.3% of the total respondents abandoned their research before publication and 43 (11.3%) of them had research publications. Only 17 (4.5%) and 6 (1.6%) of the participants received gift authorship and purchased authorship respectively (Table-II).

**Table-II:** Association between research experiences and research publications among participants.

Research experience	Total Participants n (%) (N=380)	Research publication		P- value
		Yes n= 98 (25.8)	No n= 282 (74.2)	
Research training during undergraduate/ post-graduate	178 (46.8)	78 (43.8)	100 (56.1)	<0.001
Previous research involvement	175 (46)	96 (54.8)	79 (45.1)	<0.001
Research presentations in conference	71 (18.6)	54 (76)	17 (24)	< 0.001
Research paper abandoned before publication	58 (15.2)	43 (74.1)	15 (25.8)	<0.001
Any gift authorship received	17 (4.4)	13 (76.4)	4 (23.5)	< 0.001
Any authorship purchased	6 (1.5)	4 (66.6)	2 (33.3)	0.04

Almost 62.6% of the participants considered lack of training as the main barrier in conducting research, followed by inadequate financial support (53.1%), inadequate support from institute (53.1%), inadequate support from mentors (48.1%), lack of statistical support (47.3%), insufficient time (45.5%) and difficulty in obtaining ethical approval (30.7%). All details regarding barriers of conducting research among doctors are presented in Table-III. There was statistically significant association found for inadequate financial support barrier between participants who had research publication and who did not (P < 0.001). For other barriers, there was no significant association found between participants (P > 0.05).

**Table-III:** Barriers of conducting research among doctors.

Barriers	Responses n (%)		
	Disagree	Neutral	Agree
Insufficient time to conduct research	111 (29.2)	96 (25.3)	173 (45.5)
Inadequate financial support	100 (26.3)	78 (20.5)	202 (53.1)
Inadequate support from mentor	106 (27.8)	91 (23.9)	183 (48.1)
Inadequate institutional support	105 (27.6)	73 (19.2)	202 (53.1)
Difficulty in obtaining ethical approval from the ethical review board	115 (30.2)	148 (38.9)	117 (30.7)
Lack of statistical support	91 (23.9)	109 (28.7)	180 (47.3)
Lack of research training	78 (20.5)	64 (16.8)	238 (62.6)

## DISCUSSION

This study highlights the main barriers of conducting and completion of research among doctors in Pakistan. The most common barriers were lack of training and inadequate financial and institutional support. There was a significant association found for inadequate financial support among participants who had research publications compared to those who did not. This finding emphasizes and suggests that funding support plays a crucial role in conducting and completion of research among doctors in Pakistan.

In the present study, only 25.8% of the participants had research publications and 46.1% previously involved in research projects. Similar findings were reported by Sabzwari S et al.^9^ in their study about research barrier among junior faculty of Pakistan medical universities and reported 41.5% of the faculty were involved in research. They also reported highly significant association between research involvement and research training during post-graduation. Similarly, in our study we also found significant association between post-graduate research training and research publications (< P=0.001). Few studies have also reported barriers of conducting research in Pakistan.^10^,^11^ The major barriers identified were lack of research culture, funding, deficient technology, constrained networking/collaboration, finding a mentor and lack of previous exposure to research. In our study, we also reported similar barriers. There are only a few studies available that reported research barriers in Pakistan as mentioned above. However, a few studies have identified research barriers in different settings and geographic areas.

The study was conducted to report obstacles faced by doctors in conducting research in India. They highlighted that the most frequently performed study types are case report and case control studies (observational studies).^8^ In our study, 124 (71%) of the conducted studies were observational in nature. Lack of access to research journals and absence of proper guidance or mentorship were considered as most commonly faced challenges in previous studies.^8^,^12^ In present study, 48.1% of the participants reported inadequate mentorship as barrier.

However, we reported lack of research training and financial support as most common barriers among Pakistani doctors. This disparity in findings could be due to difference in socioeconomic situations where studies were conducted. Saud AlEnazi A et al.^13^ reported the perception, barrier and research attitude among resident doctors of different speciality in Saudia Arabia. Almost 61.7% of the residents had participated in research. In our study, this proportion was reduced to 46.1% of the doctors who were involved in research. Inadequate facilities for research, lack of baseline research skills, and personal commitments were the most frequently reported barriers for conduction and completion of research projects in above-mentioned study. However, some studies have also reported institutional barriers such as lack of professional supervisor support and lack of research curriculum in the training program which were also identified as the main barriers in the present study.^12^-^14^

In this study, we only included medical doctors. Some studies have also identified research barriers among other health professionals.^15^-^17^ However, our results are comparable with previous studies. Al-Yateem N et al.^15^ evaluated research barriers among nurses in United Arab Emirates (UAE) and reported lack of time as most important barrier. Similarly, another study reported that 32.5% and 39.1% of the dentists and dental students identified lack of time and lack of supervision as the most common barriers towards scientific research in Saudia Arabia.^17^ The findings of our study are consistent with the previous literature. We also identified lack of time (45.5%) and inadequate support from mentor (48.1%) as leading barriers among doctors. Similar barriers were reported by other studies as well.^18^-^21^ This study clearly highlights and identifies the research barriers among doctors in Pakistan, which will be a first step to promote and nurture research culture in Pakistan.

### Limitations:

First, it was a cross-sectional study. Therefore, it was highly dependent on given responses and preventing the determination of causality. The small sample size may limit the generalizability or external validity of our findings. This survey was only based on identifying the factors and did not assess the possible solution for practical implication.

## CONCLUSION

According to our findings, a high proportion of doctors were previously involved in research. The main barriers in the conduction and completion of research were lack of training, financial and institutional support. It is crucial to make policies at institutional and national level to overcome these barriers to promote medical research in Pakistan especially among doctors.

### Recommendations:

It may be useful for future studies to focus on the solutions to overcome reported research barriers. Therefore, we recommend large scale, qualitative and national surveys on the similar topic to explore research barriers in depth and find out the possible solutions and practical implications. It will help to promote good quality research conduction and completion in Pakistan. However, this survey will serve as a frame work for all future studies on similar topic.

### Authors’ Contribution:

**ZAK and MJ:** Conceptualized the idea. Analyzed the data.

**AK, UM and KA:** Literature search**,** Collected the data. Revised the manuscript.

**ZAK and UM:** Prepared the first draft of manuscript.

All authors have approved final version and are responsible and accountable for the integrity of the study.

## References

[1] Murillo H, Reece EA, Snyderman R, Sung NS (2006). Meeting the challenges facing clinical research:solutions proposed by leaders of medical specialty and clinical research societies. Acad Med.

[2] Somkin CP, Altschuler A, Ackerson L, Geiger AM, Greene SM, Mouchawar J (2005). Organizational barriers to physician participation in cancer clinical trials. Am J Manag Care.

[3] Amerson RM, Strang CW (2015). Addressing the challenges of conducting research in developing countries. J Nurs.

[4] Ahmad S, Ahmad S, Ahmad U, Cheema HA, Iqbal N, Shahid A (2022). An assessment of publishing practices and barriers faced by medical students to conduct research:A cross-sectional study from Pakistan. Health Sci. Rep.

[5] Raza F, Nisa Q (2017). Perception, Attitudes and Barriers in undergraduate medical students toward medical research at rehman medical college, Peshawar, Pakistan. Khyber Med Univ J.

[6] Subhani MI, Osman A, Nayaz M (2017). Role of higher education commission (HEC) in promoting research output in Pakistan. EUrASEANs.

[7] Alrashidi YA, Alrabai HM (2021). Barriers to conduction or completion of research projects among orthopedic surgeons in Saudi Arabia. J Musculoskelet Res.

[8] Shanmukhappa SC, Abraham RR, Venkatesh VS, Abraham RR (2020). Motivators and barriers to research among doctors in the Indian medical scenario:A cross-sectional study from Karnataka, India. J Prim Health Care.

[9] Sabzwari S, Kauser S, Khuwaja AK (2009). Experiences, attitudes and barriers towards research amongst junior faculty of Pakistani medical universities. BMC Med Educ.

[10] Afzal A, Saleem H, Iqbal A, Akram A (2024). The barriers to meaningful research in Pakistan and the way forward. Anaesth Pain Intensive Care.

[11] Tirupakuzhi Vijayaraghavan BK, Gupta E, Ramakrishnan N, Beane A, Haniffa R, Lone N (2022). Barriers and facilitators to the conduct of critical care research in low and lower-middle income countries:a scoping review. PLoS One.

[12] Al Absi DT, Yousuf K, Aljaberi K, AlBreiki R, Simsekler MC, Omar MA (2024). Barriers Preventing Medical Trainees from Active Participation in Research Activities. J Multidiscip Healthc.

[13] Saud AlEnazi A, Alamri AS, AlGhamdi AS, Almansour AH, Rubaian NF, Al-Otaibi FK (2021). Perceptions, barriers, and attitudes toward research among in-training physicians in Saudi Arabia:A multicenter survey. Sci. Prog.

[14] Koelkebeck K, Stefanovic MP, Frydecka D, Palumbo C, Andlauer O, Riese F (2019). Barriers and facilitators to conducting research by early career psychiatrists:a literature review. Glob Psychiatry.

[15] Al-Yateem N, Griffiths J, McCreaddie M, Robertson-Malt S, Kuzemski D, Mathew Anthony J (2019). A national scoping study on barriers to conducting and using research among nurses in the United Arab Emirates. Policy Politics. Nurs Pract.

[16] Corrales-Reyes IE, Villegas-Maestre JD, Valdés-Gamboa L, García-Raga M, Véliz-Concepción OL, Torres-Fernández LS (2023). Factors associated with interest in scientific research in dental students of six Cuban universities. Front Educ.

[17] Abdulrahman S, Aboalshamat K, Muthana M, Sait G, Bantan N, Hafiz S (2020). Knowledge, attitude, practice, motives and barriers towards scientific research among dentists and dental students in Saudi Arabia. Open Dent J.

[18] Khalaf AJ, Aljowder AI, Buhamaid MJ, Alansari MF, Jassim GA (2019). Attitudes and barriers towards conducting research amongst primary care physicians in Bahrain:a cross-sectional study. BMC Fam Pract.

[19] Ichsan I, Wahyuniati N, McKee R, Lobo L, Lancaster K, Redwood-Campbell L (2018). Attitudes, barriers, and enablers towards conducting primary care research in Banda Aceh, Indonesia:a qualitative research study. Asia Pac Fam Med.

[20] Persad S, Mehra S, Harry V (2023). Attitudes and Perceived Barriers to Research Among Residents in Obstetrics and Gynecology in Trinidad and Tobago:A National Multicenter Cross-sectional Study. J Med Educ Curric Dev.

[21] Beshyah S, Ali K, Mustafa H, Hajjaji I, Hafidh K, Abdelmannan D (2020). Doctors'attitude and engagement in research:A survey from two emerging regions. Ibnosina J Med Biomed Sci.

